# Endocrine Disrupting Chemicals and Type 1 Diabetes

**DOI:** 10.3390/ijms21082937

**Published:** 2020-04-22

**Authors:** Barbara Predieri, Patrizia Bruzzi, Elena Bigi, Silvia Ciancia, Simona F. Madeo, Laura Lucaccioni, Lorenzo Iughetti

**Affiliations:** 1 Pediatric Unit, Department of Medical and Surgical Sciences of the Mother, Children and Adults-University of Modena and Reggio Emilia, Largo del Pozzo, 71-41124 Modena, Italy; elena.bigi@gmail.com (E.B.); iughetti.lorenzo@unimore.it (L.I.); 2 Post Graduate School of Pediatrics, Department of Medical and Surgical Sciences of the Mothers, Children and Adults—University of Modena and Reggio Emilia, Largo del Pozzo, 71-41124 Modena, Italy; silvia.ciancia.18@gmail.com; 3 Pediatric Unit, Department of Pediatrics—AOU Policlinic of Modena, Largo del Pozzo, 71-41124 Modena, Italy; bruzzi.patrizia@aou.mo.it (P.B.); madeo.simona@aou.mo.it (S.F.M.); 4 Neonatal Intensive Care Unit, Department of Medical and Surgical Sciences of the Mother, Children and Adults-University of Modena and Reggio Emilia, Largo del Pozzo, 71-41124 Modena, Italy; laura.lucaccioni@unimore.it

**Keywords:** type 1 diabetes, non-obese diabetic (NOD) mice, endocrine disruptors, bisphenol A, pesticides, phthalates, polychlorinated biphenyls, polyfluorinated substances

## Abstract

Type 1 diabetes (T1D) is the most common chronic metabolic disease in children and adolescents. The etiology of T1D is not fully understood but it seems multifactorial. The genetic background determines the predisposition to develop T1D, while the autoimmune process against β-cells seems to be also determined by environmental triggers, such as endocrine disrupting chemicals (EDCs). Environmental EDCs may act throughout different temporal windows as single chemical agent or as chemical mixtures. They could affect the development and the function of the immune system or of the β-cells function, promoting autoimmunity and increasing the susceptibility to autoimmune attack. Human studies evaluating the potential role of exposure to EDCs on the pathogenesis of T1D are few and demonstrated contradictory results. The aim of this narrative review is to summarize experimental and epidemiological studies on the potential role of exposure to EDCs in the development of T1D. We highlight what we know by animals about EDCs’ effects on mechanisms leading to T1D development and progression. Studies evaluating the EDC levels in patients with T1D were also reported. Moreover, we discussed why further studies are needed and how they should be designed to better understand the causal mechanisms and the next prevention interventions.

## 1. Background

Type 1 diabetes (T1D) is the most common chronic metabolic disease in children and adolescents. In children under 15 years, the worldwide estimated prevalent cases were 600,900 and the incident case 98,200/year [1]. The national incidence of T1D is different from state to state, varying from 1.0/100,000 people/year in China [2] to more than 60/100,000 people/year in Finland [3]. The estimated annual increase of T1D incidence in childhood is documented to be +3.4% in Europe [4] and +2.8% worldwide [5]. In Italy the T1D incidence rate is 12.26/100,000 people/year with geographical variations and the increasing temporal trend is +2.94% per year. The incidence increased from 6.0/100,000 in the 0–2 age group to 12.01/100,000 in the 3–5 years one, with a 15.75/100,000 peak in patients aged 9–11. Moreover, the risk for T1D was significantly higher in boys (13.13/100,000) than in girls (11.35/100,000) [6]. Our Italian data were similar to the worldwide data [5]. T1D incidence, treatment, and metabolic control data in low income countries are not univocal [7]. Among African populations a lower incidence is reported than in Western populations, varying from 1.5/100,000 people/year in Tanzania to 10.1/100,000 people/year in Sudan [8] with a difference age associated as recently reported in Ghana where the T1D incidence in children 0–9 yeas-old decreased from 5.1% in 1992–1994 to 3.6% in 2016–2018, while increased among adolescents (from 35.4% in 1995–1997 to 63.2% in 2016–2018) [9]. The dissimilar T1D incidence between countries and ethnic groups is probably due to differences in the genetic susceptibility and environmental factors exposure [10]. 

T1D onset results from the destruction of the pancreatic β-cells due to both cellular infiltration [11,12] and autoimmune responses [13]. Serological islet autoantibodies are directed against glutamic acid decarboxylase 65 (GAD), tyrosine phosphatase-like insulinoma antigen 2 (IA2), insulin (IAA), and β-cells-specific zinc transporter 8 (ZnT8) [14,15]. The autoimmune process against β-cells seems to be also determined by environmental triggers [16] whose potential role was supported by the low concordance of T1D incidence among monozygotic twins [17] and by the rise of T1D incidence in industrial countries. Moreover, it is nowadays known that in genetically susceptible subjects who migrate from low to high risk areas for T1D the disease incidence tends to increase; it seems acceptable that immigration is associated with exposure to new environmental factors contributing to the development of T1D [18,19].

Research on environmental factors involved in the etiology of islet autoimmunity were focused on the role of viruses [20,21], early infant diet [22,23], childhood weight gain [24], vitamin D [25], gut microbiome [26,27,28], and environmental endocrine disrupting chemicals (EDCs) [29,30,31].

EDCs are a group of exogenous compounds with high heterogeneity that are globally distributed and may be found naturally in living organisms or synthesized industrially. They could promote the autoimmunity and increase the β-cells susceptibility to autoimmune attack. Human and animal studies evaluating the potential role of exposure to EDCs on pathogenesis of T1D showed contradictory results 

In 2015, the Endocrine Society defined the EDCs as “an exogenous chemical or mixture of chemicals, which interferes with any aspect of hormone action” [32]. They include synthetic chemicals used as industrial solvents/lubricants and their byproducts: polychlorinated biphenyls (PCBs), polyfluorinated substances (PFASs), polybrominated biphenyls (PBBs), dioxins, pesticides such as methoxychlor, chlorpyrifos, dichlorodiphenyltrichloroethane (DDT), dichlorodiphenyldichloroethylene (DDE), plastics like bisphenol A (BPA), plasticizers (phthalates), fungicides (vinclozolin), and the diethylstilbestrol (DES) among pharmaceutical agents [33].

The attention of scientific community during the last decades allowed great progress in understanding the relationship between EDCs and human endocrine and metabolic diseases. Despite current knowledge, further data should be obtained and, to date, it is clear that preventive measures in EDCs exposure must be applied to avoid the impairment of human health [34]. Recent data from the LIFE PERSUADED project on phthalate exposure in Italian children and adolescent demonstrated that boys were more exposed than girls to bis(2-ethylhexyl)phthalate (DEHP) main metabolites and that children aged 4–6 years had higher median levels than those 7–10 and 11–14 years old [35].

EDCs have a non-monotonic (U-shaped or biphasic) response, so very low levels of exposure may have a stronger disrupting effect on the human body than higher doses [36,37]. The clinical effects of EDCs exposure may be manifested many years after [38] and the exposure during developmental periods (i.e., in utero, infancy, and puberty) may have detrimental effects on health predisposing subjects to several diseases [39,40]. Finally, it must be taken into consideration that it is really difficult to define a clear cause-and-effect relationship between a specific EDC and a disease. People are exposed to a “EDCs cocktail” since environmental EDCs interact with each other in an additive or synergistic way [41].

Although EDCs are able to interfere with the endocrine system, to date it is clear that they may have negative effects also on metabolic health inducing the immune system dysfunction [42,43,44].

The increased incidence of T1D cannot be explained only by genetic predisposition and the widespread use of chemicals increased together with the rise in the T1D incidence. In 2015, the Parma Consensus Statement [45] proposed that the exposure to EDCs during the development period could increase the susceptibility to diabetes and to other metabolic diseases later in life, but further research is needed to better define their role as metabolic disruptors in T1D.

**Figure 1:**
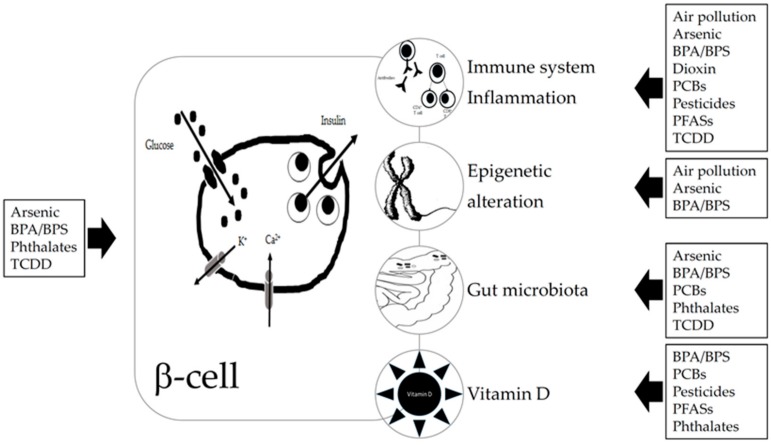
Relationship suggested between endocrine disrupting chemicals (EDCs) and type 1 diabetes (T1D) and suggested mechanisms. EDCs can act directly on beta or immune cells, by binding to receptors (aryl hydrocarbon receptor, peroxisome proliferator-activated receptors, estrogen receptors, and other ones). EDCs can affect the gut microbiota which in turn also interact with the immune system. Some EDCs have been shown to induce epigenetic changes and to impair vitamin D status. Abbreviations: BPA—bisphenol A; BPS—bisphenol S; PBCs—polychlorinated biphenyls; PFA—polyfluorinated substances; TCDD—2,3,7,8-tetrachlorodibenzo-p-dioxin.

We summarize experimental and epidemiological studies on the potential role of exposure to EDCs in the development of T1D. Literature analysis was focused on arsenic, BPA, dioxin, pesticides, phthalates, PCBs, PFASs, and air pollution. We highlight what we know on the really complex relationship between EDCs exposure and the increased risk of T1D development and why future research is needed to better understand the cause-and-effect mechanisms and the next preventive interventions. Relationships between EDCs and T1D and suggested mechanisms are summarized in Figure 1.

## 2. Materials and Methods

We retrieved relevant original cross-sectional, cohort, case-control, or nested case-control studies investigating the association between EDCs and T1D, without any restriction on setting or language. The articles were systematically searched on February 22nd 2020 in the MEDLINE database (PubMed) using the following search terms: NOD mice OR type 1 diabetes AND air pollution OR arsenic OR bisphenol* OR DDT OR DDE OR dioxin OR endocrine disrupt* OR perfluorin* OR persistent organic pollut* OR pesticide* OR polychlorinated biphenyl* OR phthalate*. The MEDLINE search was also performed adding (AND) the terms immunomodulation OR gut microbiota OR vitamin D to previous search terms associations. Moreover, a hand-screening of all the reference lists included in papers was performed to identify studies missed in the primary search process. Conference abstracts and qualitative studies (i.e., interviews, letters) were discarded. Three of the authors (B.P, P.B, and L.I.) scored the retrieved titles and abstracts independently. Subsequently, full texts of all potentially relevant papers were reviewed and were included in this review if ECD exposure levels were examined in relation to T1D incidence, age of T1D development, T1D-related autoantibodies, or T1D environmental triggers. In vivo laboratory studies in a non-obese diabetic (NOD) mouse model and in mice treated with multiple low dose administrations of toxin streptozotocin (STZ) were included and briefly described because they are important to establish the causal relationships and the mode of action. Studies on type 2 diabetes (T2D), insulin resistance, overweight/obesity, and in vitro studies were excluded.

**Figure 2:**
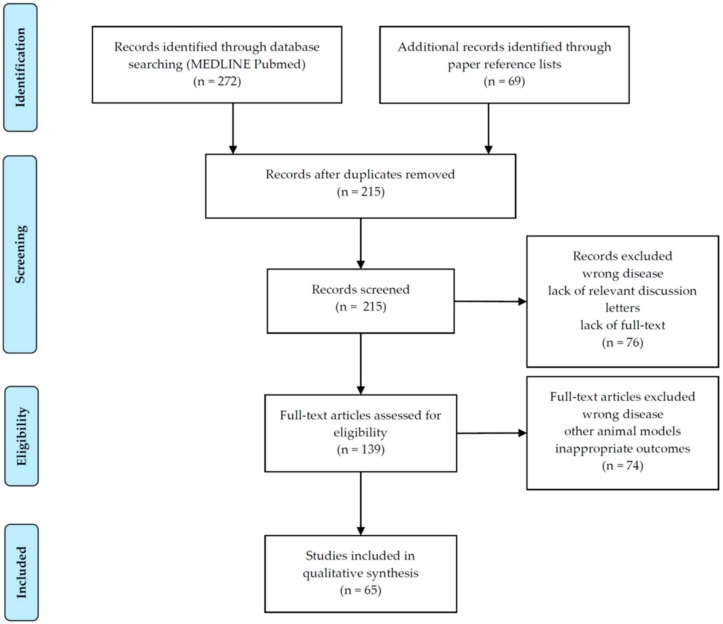
Selection of studies according to PRISMA flow diagram [46].

Overall, 215 papers were initially identified. After the screening of titles and abstracts, a total of 150 articles were discarded, leaving 65 articles to be analyzed. Full-text assessment of these articles was available for all eligible articles. Figure 2 displays the process of selection of studies.

## 3. Human and Animal Studies

### 3.1. Bisphenol

BPA is one of the most abundantly and globally produced chemicals and, considering its high rate of detection in human urine, we can assume that everyone is continuously exposed to this EDC [47]. Since the 1960s BPA has been used in the production of common containers that store food and beverages, children’s toys, and sealants in dentistry. It is rapidly metabolized to its non-bioactive forms and it has a short half-life (4–5 h). BPA use for production of polycarbonate baby bottles has been prohibited since 2011 and, under the European Plastics Regulation (EU) No. 10/2011, it is authorized to be used as a monomer for the production of plastic with a specific migration limit of 0.6 mg/kg of food [48].

#### Animal Studies

Exposure to high levels of BPA from conception throughout life increased the spontaneous development of T1D in a NOD mouse model [49,50,51]. This accelerated diabetes development was related to the impairment of macrophage function. Specifically, BPA acts by reducing macrophages’ phagocytosis that in turn leads to a decreased clearance of apoptotic cells in the pancreas and, consequently, to an accelerated insulitis development.

BPA was also demonstrated to modulate immune responses in lymphoid tissue in mice and to impair islet morphology and β-cells function in isolated rat pancreatic islets [52].

BPA treatment increases diabetes incidence and affects T-cell immunity in a multiple low-dose STZ-induced autoimmune mouse T1D model [53]. The diabetogenic mechanism was different according to dose exposure. Low-dose BPA exposure in the early stage of T1D development might cause a significant decrease in T-cells along with a trend toward increased expression of pro-inflammatory cytokines. High-dose BPA exposure was not accompanied by perturbations in T-cells levels, but there was a significant impact on pro-inflammatory cytokine levels.

Another study in a STZ-induced T1D mice model evaluated the functional change of insulin secretion and glucose homeostasis in the pancreas by administration of BPA. BPA alone treatment affected calcium homeostasis, glucose level, and plasma insulin level, but BPA + STZ treatment aggravated calcium homeostasis, endoplasmic reticulum stress, and apoptosis of pancreatic cells [54].

More recently, Xu et al. [55,56] reported that in NOD mice T1D development was altered by BPA according to the window of exposure and sex.

Finally, bisphenol S (BPS) is a common replacement for BPA in plastics and its continuous use resulted in a widespread exposure. Only one recent study in NOD mice evaluated if BPS can alter T1D risk and glucose homeostasis [57]. Results suggest that BPS has sex- and diet-dependent effects and, with respect to BPA, it involves immunomodulation but also other mechanisms of action, such as gut microbiota and epigenetics.

#### Human Studies

The first and only epidemiological study we found on BPA and T1D was published in 2018 by İnce et al. [58]. Urinary BPA levels were evaluated in 50 Turkish children with T1D and 50 matched healthy subjects. Although values were not statistically significant, due to the small number of recruited patients, T1D patients had higher mean urinary BPA concentrations compared to controls (27.71 ± 15.53 μg/g vs. 25.37 ± 17.89 μg/g creatinine, respectively). Moreover, an inverse significant correlation between urinary BPA levels and birth weight was found in T1D children and it was demonstrated that the use of plastic kettles and the consumption of dairy products in plastic boxes significantly increased the urinary BPA concentrations. Considering the cross-sectional design of the study, it was not possible to explain the mechanism underlying the relationship between urinary BPA levels and T1D. Surely, another limit of this study was that it did not distinguish between new-onset versus existing T1D.

Although T2D is not the topic of this review, it might be useful to remember that Lang et al. [59], analyzing BPA concentrations and health status in 1455 U.S. adults from the National Health and Nutrition Examination Survey (NHANES) 2003–2004, reported that higher urinary concentrations of BPA were associated with fasting glucose and insulin levels and homeostatic model assessment index. Authors hypothesized that exposure to BPA could be related to T2D, which is mainly characterized by insulin resistance and high body mass index. We need to pay attention to the occurrence of abdominal adiposity and metabolic syndrome also in patients with T1D [60]. 

In conclusion, human studies supported mainly a role of BPA exposure in the development and progression of insulin resistance and this knowledge must be taken into consideration in the management of patients with T1D. However, animals’ immune and inflammatory responses to bisphenol support the idea that further research focusing on the relationship between BPA/BPS exposure and T1D is still needed in humans, to better understand their potential health consequences and the cause-and-effect link.

### 3.2. Persistent Organic Pollutants

Persistent organic pollutants (POPs) include several chemicals accumulating in humans. Dioxins, PCBs, pesticides, and certain brominated flame-retardants are lipophilic, are stored in adipose tissue after ingestion by food chain, and have a long half-life (from months to years). PFASs, including perfluorooctane sulfonate (PFOS), perfluorooctanoic acid (PFOA), perfluorohexane sulfonate (PFHxS), perfluorononaoic acid (PFNA), perfluorodecanoic acid (PFDA), and perfluoroundecanoic acid (PFUnDA), are emerging POPs. They have attractive water and oil repellent properties; they are widely diffused being used in industrial and consumer products since the 1950s [61]. People are exposed to PFASs daily by contaminated food, water, and air, independently to industry nearby [61,62]. They are stored in the liver and have a really long half-life (from months to years) according to the length of their carbon chain.

#### 3.2.1. Pesticides

DDT is a synthetic insecticide with a long half-life, extensive use, and lipophilic nature. The U.S. banned DDT in 1972 due to its effects on the environment and human health. Even if DDT is banned from the market it is still present in the environment. Moreover, in some low-income countries it is still widely used. People are most likely to be exposed to DDT from foods and it can be absorbed by breathing or by touching contaminated products. In the body, DDT is converted into several breakdown products called metabolites, including the metabolite DDE. Both chemicals are stored in fatty tissue. 

##### Animal Studies

Published data on the effects of DDT and DDE in animal models are not univocal because both immunosuppressive and immunoproliferative properties were demonstrated [63,64].

Cetkovic-Cvrlje et al. [65] for the first time demonstrated that chronic high doses of DDE significantly increased the T1D incidence and the severity of hyperglycemia in NOD mice. The immunomodulatory mechanism of action was related to a decrease in regulatory T-cells activity and a suppression of secretion of protective cytokines, such as interleukin (IL)-4 and IL-10. Conversely, an acute high dose of DDE promoted the pathogenic immune response enhancing T-cells proliferation, proinflammatory, and T-helper (Th) 1-type cytokine secretion.

##### Human Studies

In a Swedish case-control study, including 150 children with T1D diagnosis and 150 controls matched for age and day of birth, the levels of p.p’-DDE were measured in stored serum of mothers during pregnancy. The p.p’-DDE concentrations were comparable between T1D cases and controls (9.2 vs. 9.6 ng/mL, respectively) and decreased over years. The authors did not support the involvement of p.p’-DDE as a trigger for the development of T1D [66]. 

The DDE levels were measured in two birth cohorts from the FINDIA and DIABIMMUNE studies and no association between this insecticide and T1D or autoimmunity was found [67].

A study performed in Egypt found that 75 children with newly diagnosed T1D had significantly higher serum levels of eight out of nine studied organochlorine and organophosphorus pesticides compared to healthy controls [68]. The lindane was the most common organochlorine pesticide found in T1D patients (70.7%) followed by o.p’-DDD (21.3%), p.p’-DDE (21.3%), and endrin (10.7%). The most common organophosphorus compound detected was malathion (65.3% of cases). The highest odds ratio was found in these compounds, suggesting that exposed children have an increased risk of T1D.

Impairment of β-cells function and poor glycemic control were related to pesticide exposure. The longitudinal association between urine and serum pesticide levels and risk factors for cardiovascular disease was recently evaluated in 87 U.S. youths with diabetes (57.5% with T1D). Patients with the highest levels of 2,4-D, 4-nitrophenol and trans-nonachlor had higher HbA1c levels and lower fasting C-peptide levels. These data suggested that exposure to select pesticides may be associated with increased risk for cardiovascular disease [69].

In conclusion, we found contradictory results and other studies are needed to clarify the relationship and potential causal pathways between T1D and pesticides. Data on animal models might not be directly extrapolate to humans, so it is necessary a greater awareness on the potential effects of DDE on human health. On the other hand, studies recommend a careful approach before reintroducing these pesticides to prevent some disease.

#### 3.2.2. Polychlorinated Biphenyls

PCBs are a group of very stable synthetic chemical mixtures, widely used in electrical equipment, ink solvents, and plasticizers until the 1979, when the U.S. Environmental Protection Agency banned their use.

##### Animal Studies

In NOD mouse models, we found only one experimental study evaluating the effects of PCB-153 exposure on T1D development [70]. The study showed that both high and low levels of PCB-153 exposure reduced the incidence of T1D due to a significant immunosuppression characterized by a decrease in T helper type T-cells along with insufficient T-cell function and decreased IL-2 secretion. Moreover, results bring awareness about the potential interaction of PCB-153 with other co-existing pollutants and its potential masking effects on the other agents’ consequences on immune system.

##### Human Studies

The first study evaluating PCB levels in humans with diabetes was published by Longnecker et al. [71]. In this small U.S. study, it was found that in 44 pregnant women with diabetes (primarily T1D) the median serum PCBs level was 3.77 mg/L, 30% higher than in controls (*n* = 2201). Although authors demonstrated that the odds ratio for diabetes increased concurrently to the increasing of PCB levels, the causal relationship was not clearly determined since the study was cross-sectional. 

Levels of T1D autoantibodies are generally increased in genetically predisposed people and their development may be accentuated by EDCs. The immunomodulatory long-term effect of PCBs was firstly described by Langer et al. [72]. Although the PCBs levels were not measured and the prevalence of T1D could not be determined due to the retrospective design of the study, the authors reported a possible relationship between PCBs and the prevalence of GAD that was four times higher in 240 employees of a factory producing PCBs in East Slovakia compared to 704 subjects from other less polluted areas of East Slovakia. 

Finally, in the Swedish study the levels of PCB-153 were also measured. The serum maternal median concentrations were high in both case and control groups being 2.4 vs. 2.6 ng/mL, respectively, and the exposure levels resulted significantly decreased from 1970 to 1990. This in utero exposure was not correlated with a higher risk of T1D development in the offspring. Contrary to what was assumed, the estimated risk went in the opposite direction raising the unbelievable question whether POPs exposure may have a protective effect. The authors speculated that studied POPs might act as indicators for polyunsaturated fatty acids which have anti-inflammatory effects and thus might protect against the T1D risk [66].

Again, epidemiology and animal studies provide few and inconclusive data about the link between PCBs exposure and development of T1D. Both positive and negative associations were reported. A nested case-control study and a NOD study investigated the role of PCB-153 in T1D and suggested that these compounds might have a possible protective effect, rather a negative one. More studies are needed to better understand the relationship between these POPs and T1D.

#### 3.2.3. Polyfluorinated Substances (PFAS)

PFASs are a group of man-made chemicals that have been used since the 1940s to get fluoropolymer coatings and a wide range of products resistant to heat, oil, stains, grease, and water. Concerns for these chemicals are due to their capacity to persist in the environment and to bio-accumulate in food chains. In humans PFASs have long half-lives (years) and are mainly stored in the liver, as people are daily exposed to contaminated food, water, and air, independently to industry nearby. Exposure may also occur by using products containing PFASs. 

##### Animal Studies

We found only one study investigating the effect of PFAS on T1D development in the NOD mouse model [73]. Life-long exposure to PFUnDA had detrimental effects on the pancreatic islets, but the accelerated insulitis due to highest exposure was not accompanied with a rise of T1D development. Moreover, PFUnDA affected the immune system in a non-monotonic dose response curve considering that the low and intermediate exposure dose caused a delay of the T1D development.

##### Human Studies

Our small case-control study was the first published one that investigated the serum PFASs concentrations in 25 children and adolescents with T1D at onset. We found that young patients newly diagnosed with T1D had significantly higher levels of PFOS respect to healthy matched controls (1.53 ± 1.50 vs. 0.55 ± 0.15 ng/mL, respectively; *p* < 0.001), while PFOA levels were comparable. We suggested that exposure to PFAS, even at very low concentrations, may have detrimental effects on the immune system, which may increase the risk of T1D development [74].

In contrast to our data, a recent published paper demonstrated that in children with high genetic susceptibility to T1D, the prenatal or early childhood exposure to low levels of several POPs, including 14 PFASs, was not a risk factor for the development of β-cell autoimmunity or the progression to clinical T1D [67]. In this Finnish/Estonian study it was found that the circulating concentrations of POPs were higher in Estonian children than in Finnish ones. Looking at the national incidence of T1D for each country, this data appears controversial: the less POPs exposed Finnish population presents a higher incidence of T1D [3] than the more POPs exposed Estonian population [75]. Despite the relatively small sample, the study is relevant because the case-control cohorts were matched for age, gender, Human Leukocyte Antigen (HLA) for T1D risk, geographical location, were adjusted for breastfeeding time, and were longitudinally monitored for chemical levels and appearance of T1D-associated autoantibodies levels. 

Finally, a large cross-sectional study from the U.S. analyzed serum PFASs levels in a highly exposed population in the mid-Ohio River valley including 820 subjects with T1D [76]. PFOA levels, but not PFOS ones, were higher than in the general U.S. population [77] and both PFOA and PFOS concentrations were higher than in the Italian [74] and Finnish/Estonian studies [67]. The high levels of PFASs were significantly associated with a lower risk of T1D development both in children and adults and the authors suggested that these chemicals might be protective against the disease, since they enhance oxygenation which could protect β-cells.

In conclusion, the human studies on the role of PFASs in the development of T1D show contrasting results. Since both epidemiological and animal studies on PFASs and T1D are sparse and report contradictory findings, more studies are needed to assess the effects of exposure on human health. Moreover, analyzing data we must keep in mind the possible different effects due to high or low exposure levels, often resulting in increased T1D/autoimmunity risk and decreased T1D risk, respectively.

#### 3.2.4. Dioxin

Dioxins represent a group of chemicals compounds produced primarily during the combustion of chlorinated chemical products. They break down very slowly and their half-life is estimated to be from 7 to 11 years. Dioxins are absorbed and stored in fat tissue and accumulate in the food chain. More than 90% of human exposure is through contaminated food, mainly animal products. The most harmful dioxin is the 2,3,7,8-tetrachlorodibenzo-p-dioxin (TCDD) that was used in herbicides.

##### Animal Studies

Impairment of the glucose-stimulated insulin secretion [78] and β-cell exhaustion [79] were demonstrated in TCDD exposed animal models. 

Conversely, in the NOD mouse model, chronic treatment with TCDD completely blocked T1D development and the effect disappeared in 50% of mice when TCDD administration was stopped. The immunosuppressive effect was shown to be linked to the activation of the aryl hydrocarbon receptor [80].

However, the effect of TCDD on the immune system is complex. In mice, exposure to TCDD during in utero critical developmental stages promoted the development of autoimmune diseases later in life [81] and probably T1D could be included among them.

##### Human Studies

To the best of our knowledge, dioxins have been associated to T2D [82], but no epidemiological study investigated the relationship between dioxin exposure and T1D development.

In conclusion, due to the omnipresence of dioxins, all people have background exposure and a certain level of dioxins in the body. Considering the high toxic potential of this class of compounds, we believe that human studies need to be undertaken to understand the serum dioxin levels and its possible role in T1D development.

### 3.3. Phthalates

Phthalates are a group of chemicals used to make plastic more flexible and harder to break. Dibutyl-phthalate (DBP), DEHP, and dimethyl-phthalate (DMP) are the most common phthalates used on a large scale in habitually used products. People are exposed to phthalates by eating and drinking foods that have been in contact with containers and products containing phthalates. The half-life is short (from hours to days).

#### Animal Studies

Animal models suggested a close relationship between phthalates and metabolic disease. Exposure to phthalates leads to a reduction in the pancreatic insulin content, a loss and an abnormal ultrastructural pattern of β-cells [83].

Phthalates’ effects were reported in studies together with BPA exposure, so it is really difficult to define a clear relationship between phthalates and T1D. When the chemicals were combined, the phthalates seemed to counteract the ability of BPA to promote diabetes development, although they did not counteract insulitis development [51]. Moreover, phthalate metabolites were found to be less potent in affecting insulin secretion [84].

#### Human Studies

The first and only epidemiological cross-sectional study evaluating the relationship between phthalate exposure and T1D was performed in Portugal. Urinary levels of mono-isobutyl phthalate (MiBP), a metabolite of di-isobutyl phthalate (DiBP), were not significantly different between studied groups (children with T1D at onset, children with T1D for more than 6 months, and controls), but children with new-onset T1D had higher MiBP concentrations [85]. The sample of this study was small, and this aspect can probably explain the lack of statistical significance. Clinical studies with larger population samples should be performed to better understand the relationship between phthalate and T1D.

In conclusion, the identification of a causal link between phthalate and T1D remains controversial. Considering that phthalate may be present in considerably high concentrations in human serum, further studies evaluating the impact of phthalates on β-cell function are warranted in more sensitive cell types and model systems.

### 3.4. Arsenic

Heavy metals, mainly arsenic, in foods or water might influence the autoimmune mechanisms in genetically susceptible individuals and have been found to have a toxic effect on β-cell function [86]. Arsenic is a natural component of the earth’s crust and is widely distributed throughout the environment. It is highly toxic in its inorganic form to which people are exposed through drinking and using contaminated water, industrial processes, eating contaminated food, and smoking tobacco. Adverse health effects that may be associated with long-term ingestion of inorganic arsenic include diabetes.

#### Animal Studies

MicroRNAs were demonstrated to play a crucial role in the pathogenesis of autoimmune diabetes [87]. Recently, Ramdas et al. [88] reported a significant increase in miR-2909 expression in NOD mice compared to control mice. The authors concluded that exposure to low doses of arsenic can regulate miR-2909 expression in the pancreatic β-cell which in turn control the expression of genes coding for pancreatic duodenal homeobox 1 and phosphatidylinositol-3-kinase which play an important role for the transcription, synthesis, and release of insulin by the pancreatic β-cell.

Sodium meta-arsenite, an orally available arsenic compound, was demonstrated to reduce proliferation and activation of T-cells, thus preventing autoimmune diabetes in NOD mice [89].

Moreover, expression of transporters of trivalent inorganic arsenic was found high in specific tissues in STZ-diabetic mice, with consequent increased transport and/or accumulation of arsenic and, probably, susceptibility to arsenic-induced toxicity [90].

#### Human Studies

Recently, levels of different metals were evaluated in the cord blood of 20 children who developed T1D and of 40 controls matched for age and gender, recruited in a Swedish cohort trial. Children who later developed T1D had significantly more often increased concentrations of the combination of aluminum and arsenic. These findings suggest that exposure to toxic metals during pregnancy might be one among several contributing environmental factors to the disease process if confirmed in other studies [91].

Arsenic species plasma levels were evaluated in a population-based, cross-sectional U.S. study including 429 children and adolescents with T1D. Lower levels of inorganic arsenic and higher levels of arsenic metabolites were associated with both higher odds and prevalence of T1D. Stronger associations were found in subjects who also had higher folate levels [92].

A case-control study from Newfoundland and Labrador, Canada, found that at the community level, higher levels of arsenic and fluoride in drinking water were associated with a higher incidence of T1D. Specifically, the positive association of arsenic with T1D in an area with localized high arsenic levels in groundwater and also with high rates of T1D could be considered interest [93].

In conclusion, in animal studies arsenic seems to disrupt β-cell function with consequent impairment of insulin secretion. A possible role in the development of T1D was suggested. Adverse effects on human health may be associated with long-term ingestion of inorganic arsenic; however, without more longitudinal evidence, we cannot yet say if arsenic exposure or arsenic metabolism contributes to T1D pathogenesis.

### 3.5. Air Pollution

Air pollution is a mixture of solid particles and gases in the air. It is well known that air pollution has detrimental health effects mainly on pulmonary function, but not exclusively. These effects depend by the particulate matter (PM) suspended in the air. The particles have different sizes varying from 0.001 to 100 µm and the finest ones (PM2.5) can cause alterations in the endocrine system. The PM2.5, in fact, are diffused widespread throughout the body and through the circulation they can reach endocrine glands.

#### Animal Studies

Nemmar et al. [94] studied the effect of diesel particulate on the pancreas of normal and STZ-diabetic mice. The authors demonstrated a significant increase of apopototic islet cells related to an increase of oxidative stress in STZ-diabetic mice.

On the other hand, PM2.5 can worsen the metabolic control in a T1D rat model. Rats exposed to PM had a significant impairment of glycated hemoglobin related to an increase of IL-6 and results supported the role of inflammation played by PM [95].

#### Human Studies

To evaluate the impact of PM2.5 on the occurrence of T1D Gonzáles et al. [96] reviewed data of children and adolescents with T1D at onset who received their first insulin injection between 2000 and 2007. Comparing these data with the PM rates obtained from Environmental Services they demonstrated a significant increase of relative risk related to PM2.5 showing that air pollution factors could be related to peaks of T1D incidence. 

Ozone exposure was demonstrated to be significantly higher in children with T1D respect to healthy children, suggesting that air pollution may be a predisposing factor for the development of T1D [97].

However, a systematic analysis did not clearly support the role of air pollution in T1D development and progression, while suggested a relationship between air pollutants and T2D [98].

A Scandinavian study, in a cohort of children with T1D compared to randomly selected control children, matched for HLA genotype and birth year, clearly demonstrated the importance of the windows of susceptibility. Exposure to air pollution based on nitrogen oxides, traffic density, and ozone were evaluated in all subjects. This study demonstrated that the mothers of T1D children have been more exposed to air pollutants during pregnancy than the mothers of control children [99].

Finally, a recent study suggested that air pollution exposure can have an important role in the inflammation state increase in children with T1D, contributing in turn to the micro- and macrovascular impairment [100].

In conclusion, published studies demonstrated a relationship between air pollutants and T1D development in childhood. However, because this link seems to be dependent on other different factors, like time and age of exposure, further studies are needed to better clarify the role of these factors and the use of advanced biological techniques could probably help us in understanding the air pollutants’ effects and the mechanisms involved in the onset and progression of the disease.

## 4. EDCs’ Pathogenetic Mechanisms on T1D Environmental Triggers

EDCs can accelerate T1D development by several mechanism of action on known T1D triggers, such as immunomodulation, gut microbiota, and vitamin D pathway [29]. Here we briefly report on these direct pathogenetic mechanisms.

### 4.1. Immunomodulation

Due to the demonstrated relationship between the endocrine and immune systems, it was supposed that the immune system represents one of the targets of EDCs action. A recent review summarized the current knowledge on EDCs’ effects on immune cells [101].

The disruption of immune system and cytokine levels can result in autoimmune diseases, such as T1D. The main EDCs’ mechanisms of action inducing the development of diabetes were extrapolated from animal studies and can be summarized as follows: (1) in a NOD mice-model, BPA, but not phthalates, modulates the release of cytokines like IL-4, IL-6, IL-10, tumor necrosis factor (TNF)-α, and interferon (IFN)-γ) in primary culture of splenocytes and pancreatic lymph node cells exposed to BPA [51]; (2) in STZ-induced T1D mice model, BPA could induce Ca2+ imbalance resulting in oxidative stress and leading to apoptosis of β-cells [54]; (3) BPA has dose-related diabetogenic action because low doses affect the T-cells whereas high doses modulate IFN-γ and TNF-α level [53].

Moreover, in vitro and human exposure to PFAS were demonstrated to impair cytokine secretion and to reduce regulatory T-cells or Th17 cells [102,103]. These alterations could explain a modified immune response to the viral infection and consequently the development of autoimmunity.

### 4.2. Gut Microbiota

Gut microbiota has an important role in the regulation of metabolism. Although causality between dysbiosis and T1D has not been proved yet in humans because long-term placebo-controlled trials are missing, literature data suggested a role of the gut microbiota in the disease development [27,28,104,105] and a recent published review concluded that the altered abundance of specific members or reduced diversity of gut microbiota was associated with the progression of T1D [106].

In animals, the gut microbiota composition was shown to be crucial for the development of a healthy immune system and the right composition supports oral tolerance and protects against enteral virus infections. Microbial colonization of *Bifidobacterium* was demonstrated to be lower in patients with T1D [107,108,109,110,111].

An increased abundance of *Bacteroides* characterized the microbiota of T1D children who seroconverted with respect to non-converters. Moreover, in T1D patients the amounts of *Bacteroides* were found significantly higher, whereas that of *Prevotella* were significantly lower than in healthy controls [108,112]. These data are important because *Bacteroides* were associated with gastrointestinal inflammation and increased intestinal permeability, while *Prevotella* appeared to be protective. It was hypothesized that dysbiosis of the gut microbiota could exacerbate gut inflammation before disease onset [107] and evidence already demonstrated that patients with T1D had increased intestinal permeability [113,114].

EDCs can be differently metabolized by gut microbiota, which can alter the absorption, distribution, metabolism, and excretion of these chemicals [115] with potentially different effects on health.

Exposure to EDCs may increase or reduce the growth of specific gut microbial populations as demonstrated in animal models for arsenic [116], BPA [117], mixture of diethyl phthalate, methylparaben, triclosan [118], PCBs [119], or TCDD [120]. Interaction between POPs with the gut microbiome was shown to be mediated via aryl hydrocarbon receptor activation [121].

Taken together, these few studies suggest that exposure to EDCs can impair the normal gut microbiota and potentially increases the risk for T1D development. However, the mechanisms underlying these effects remain to be elucidated.

### 4.3. Vitamin D

A deficit of vitamin D is associated with increased risk of autoimmune diseases, including T1D. Vitamin D has immunomodulatory effects and the relative molecular mechanisms as well as the clinical studies on the use of vitamin D in T1D were extensively reviewed [122,123]. A potential protective effect of vitamin D against the development of T1D was supposed and it may be due to the suppression of insulitis by modification of CD8+ and CD4+ T-cells, B lymphocytes, and macrophage infiltration [124,125].

In animal studies it was demonstrated that the exposure to POPs, mainly PCBs and DDT, impair vitamin D homeostasis and decrease serum levels of both 25-hydroxyvitamin D [25(OH)D] and 1,25-hydroxyvitamin D [1,25(OH)D] [126,127].

Epidemiological studies suggested a correlation between exposure to POPs and decrease of serum vitamin D levels. In a Spanish population-based cohort, cross-sectional study including 2031 pregnant women it was found that the higher the PCB180 levels, the lower 25(OH)D3 levels. Moreover, a non-monotonic inverse association between the sum of predominant PCB (PCB 180, 153, and 138) and 25(OH)D3 concentrations was demonstrated [128]. The first human study linking the organochlorine (OC) pesticides and serum 25(OH)D was performed by Yang et al. [129]. In 1275 adults it was found that concentrations of p,p′-DDT, p,p′-DDE, and β-esaclorocicloesano were significantly and negatively related to serum 25(OH)D levels. Exposure to these chemicals may lead to lower level of serum vitamin D and probably vitamin D deficiency. Few lines of human evidence are available on the destructive role of BPA and phthalate in the vitamin D endocrine system. Johns et al. [130] in a cross-sectional study including 4724 adults reported an inverse association between total serum 25(OH)D and both urinary levels of DEHP metabolites in the overall population and urinary BPA in women. The same negative correlation between urinary phthalate metabolites and BPA and circulating total 25(OH)D was demonstrated also in a large prospective cohort of 477 pregnant women [131]. Finally, since PFAS were associated with altered sex steroid [132] and thyroid hormone levels [133] it was hypothesized that they may also affect vitamin D metabolism. In the recent NHANES population-based, cross-sectional study, PFAS were detected in over 98% of samples obtained by 7040 children and adults. Each two-fold increase in PFOS was associated with 0.9 nmol/L lower total 25(OH)D levels and higher serum PFOS concentrations were associated with increased odds of being vitamin D deficient. In contrast, each two-fold increase in PFHxS was associated with 0.8 nmol/L higher total 25(OH)D and lower risk of being vitamin D deficient [134].

## 5. Conclusions and Perspectives

T1D is a complex disease caused by the interaction of genetic and environmental factors. Many environmental triggers have been implicated in the continuous increase of T1D incidence, but no major determinants have been clearly identified and the involved mechanisms are still not well understood. Over the last decades, evidence of an important role of EDCs in the diabetes escalation has accumulated. However, few studies with small sample sizes evaluated the exposure to EDCs in people with T1D demonstrating contradictory results and, to the best of our knowledge, no published study reported a direct association between the exposure to EDCs and the incidence of T1D. The better the knowledge on levels of exposure to EDCs, the better the understanding of the impact of EDCs on health, mainly in children. It is possible to speculate that the different exposure that was demonstrated for phthalates in children and adolescents according to gender and age, seems to go “hand in hand” with T1D incidence data and could also involve all the other EDCs.

To date, it is estimated that thousands of compounds could have endocrine-disrupting properties and the size of the EDCs group is expected to further increase. It is difficult to define the exact dose of exposure being dangerous for human health, even though we know that exposure to both very low or high doses of EDCs may have effects on health; moreover, these effects could be observed in the distant future, such as in the offspring of exposed subjects. Experimental evidence showed that EDCs can affect the immune system and the development of autoimmunity, as well as the function and survival of β-cells. However, it is really difficult to have an exhaustive understanding of the mechanisms of action because of the complex interaction between several EDCs themselves and with other environmental factors.

Clearly more comprehensive epidemiological studies are necessary. Considering that the fetal life is the most susceptible period, these studies should firstly analyze data on maternal EDCs exposure before and, mainly, during pregnancy also using the existing regional database and the national Environmental Protection Services. These data should be associated with the ones on the exposure of children with T1D, especially during the other windows of susceptibility and at onset of the disease. On the other hand, using large and long-term cohorts, the evaluation of exposure to many different EDCs over time and to a mixture, rather than to single compounds, should be included. 

Moreover, new research approaches could be developed to overcome methodological limits and to obtain risk profiles in single individuals that could be applied to multi-factor diseases. Using a multidisciplinary approach and big data platforms, an attempt could be done to verify biological variations (studied with the various “omics”) related to a disease or a risk of disease.

These are methods of great interest that hopefully will provide the clinician with tools to make decisions in line with the concept of personalized medicine. However, they need to be further refined and made more precise and sensitive. While waiting for this to happen, it is strongly advisable to adopt a precautionary principle and to put in place all possible measures to control, prevent, and limit contacts with EDCs, in particular during the first 1000 days of life which represent the period of greater sensitivity and more at risk for adult life.
